# Intervention Mapping to Adapt Evidence-Based Interventions for Use in Practice: Increasing Mammography among African American Women

**DOI:** 10.1155/2015/160103

**Published:** 2015-10-26

**Authors:** Linda Highfield, Marieke A. Hartman, Patricia Dolan Mullen, Serena A. Rodriguez, Maria E. Fernandez, L. Kay Bartholomew

**Affiliations:** ^1^Department of Management, Policy and Community Health, School of Public Health, University of Texas, 1200 Pressler Street, Houston, TX 77030, USA; ^2^Department of Health Promotion and Behavioral Sciences, School of Public Health, University of Texas, 7000 Fannin Street, Houston, TX 77030, USA

## Abstract

This paper describes and demonstrates the use of the systematic planning process, Intervention Mapping, to adapt an evidence-based public health intervention (EBI). We used a simplified version of Intervention Mapping (IM Adapt) to increase an intervention's fit with a new setting and population. IM Adapt guides researchers and practitioners in selecting an EBI, making decisions about whether and what to adapt, and executing the adaptation while guarding the EBI's essential elements (those responsible for effectiveness). We present a case study of a project in which we used IM Adapt to find, adapt, implement, and evaluate an EBI to improve mammography adherence for African American women in a new practice setting in Houston, Texas. IM Adapt includes the following (1) assess needs and organizational capacity; (2) find EBIs; (3) plan adaptations based on fit assessments; (4) make adaptations; (5) plan for implementation; and (6) plan for evaluation of the adapted EBI. The case study shows an example of how public health researchers and practitioners can use the tool to make it easier to find and use EBIs, thus encouraging greater uptake. IM Adapt adds to existing dissemination and adaptation models by providing detailed guidance on how to decide on effective adaptation, while maintaining the essential elements of the EBI.

## 1. Background

Using evidence-based interventions (EBIs) to improve the health of the public improves the likelihood of program effectiveness and saves resources used in “reinventing the wheel” to address a particular health problem [1]. An evidence-based intervention (EBI) (including programs, policies, or practices) is one that has been shown to be effective through the application of sound scientific testing. Population impact on health is determined not only by the effectiveness of specific interventions, but also by how widely they are used. Governments have worked to improve opportunities for scale-up of EBIs. Nevertheless, uptake of EBIs is less than optimal and barriers to use are significant [2–4].

Challenges to using EBIs in practice include finding EBIs and their materials and deciding whether and how to adapt for a new setting. Planners need to assure a good match between the EBI and the new setting's capacity, health problem, context, and the at risk population [5, 6]. Furthermore, practitioners need to carefully consider whether to make changes in an EBI. Even small adaptations in the EBI are not trivial since adaptations may harm essential elements (also known as core elements or active ingredients) that made the EBI effective. Therefore, when adaptation is necessary to improve program fit, planners must determine not only whether or not a program works, but also which essential elements make the program successful [7, 8]. Unfortunately, program evaluations rarely report on which features of a program constitute these “essential elements.” Because separate intervention elements are not usually tested independently, new users may not be able to identify and thereafter protect essential elements [9].

If planners find an EBI with reasonable fit, but decide adaptation is needed, a systematic approach can help them retain the balance between fidelity to original program design and adaptation to improve fit [7, 10–13]. For example, Lee and colleagues assess differences between the new population and the original population, execute content adaptation and pretesting, and plan an evaluation of an adapted EBI. Van Deale and colleagues [13] present a framework for a high level of community involvement to implement the essential elements of a program with fidelity while still allowing for adaptation to fit the needs of the new population or setting. The authors also advise planners to use Intervention Mapping as a way of identifying and articulating the essential elements of a program that should be maintained and implemented with fidelity. Intervention Mapping provides a systematic approach that adds detailed “how tos” to existing frameworks.

In this report, we describe Intervention Mapping to adapt EBIs for use in practice (IM Adapt) and present a case study that used Intervention Mapping to find, adapt, implement, and evaluate an EBI. We used this systematic approach in a community-based project to improve mammography rates for African American women in a mobile mammography practice setting in Houston, Texas [14]. Epidemiologic research has shown that African American women are less likely to use mammography screening [15–17] and more likely to miss scheduled mammography appointments [18] and to be diagnosed at a later stage of breast cancer [18] than their Caucasian counterparts.

## 2. Methods

### 2.1. Case Study

In the project we found, adapted, implemented, and evaluated an EBI to help underserved African American women in Houston, Texas, keep appointments for mammography screening. The needs assessment and basic program search are described elsewhere [19], as are the evaluation results [20]. The largest mobile mammography provider in the Houston area served as the new implementation setting.

### 2.2. IM Adapt: Intervention Mapping for Adaptation

**Figure 1:**
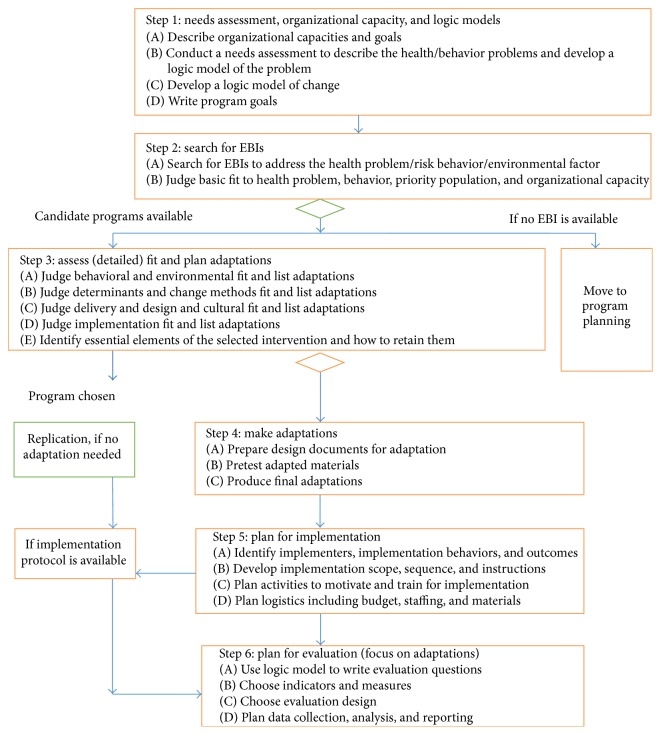
The IM Adapt Framework to adapt evidence-based interventions for use in practice.

For this community project we used a modified version of Intervention Mapping to guide the steps and tasks for adapting and implementing an evidence-based program [21–23]. Intervention Mapping is a systematic approach for developing theory- and evidence-based health promotion interventions that consists of six steps.  Figure 1 presents a simplification of Intervention Mapping to help planners compare candidate EBIs to their community program needs and adapt when necessary [23, 24]. From an Intervention Mapping perspective, systematic adaptation requires that planners make adaptation decisions by comparing the logic of change in the EBI with the needs of the new community. Planners should only make changes that correspond with mismatches between the EBI and community needs.

The case study applied the steps of IM Adapt: (1) conduct a needs assessment and assess organizational capacity; (2) search for EBIs; (3) assess fit and plan adaptations; (4) make adaptations; (5) plan for implementation; and (6) plan for evaluation with a focus on changes to the EBI (see Figure 1).

**Step 1 (conduct a needs assessment and assess organizational capacity).**

The first step of adapting an EBI following IM Adapt is to fully understand the health problem in the new site. The planning group completes four tasks: (1A) assess organizational capacity; (1B) conduct a needs assessment and develop a logic model of the problem; (1C) develop a logic model of change; and (1D) write program goals for expected outcomes from implementing the EBI at the new site. The logic model of the problem illustrates how risk behaviors and environmental factors are causally related to the health problem. Following the creation of the logic model of the problem, planners transit to a logic model of change to describe desired change. Planners show how theory-based change methods are proposed to influence first the determinants of behavior and environment, then the behavior and environmental factors, and finally the health problem and quality of life.

**Step 2 (search for EBIs).**

The second step involves two tasks: (2A) search for an EBI and (2B) judge basic fit to identify interventions to review in more detail in Step 3. Basic fit is an initial assessment of how well an intervention tested in one setting might fit the needs and resources in another setting.

In general, planners would first be looking for an EBI described in a web-based database of interventions [25, 26]. Planners may want to review websites with evidence-based strategies derived from systematic reviews to gain a broad view of effective interventions. Since these usually provide descriptions of general approaches (e.g., one on one education), these may require more effort to obtain specific interventions and materials [27–29].

To judge basic fit of the EBIs identified, the planner considers whether the focus of the EBI matches the health problem, behaviors, environmental conditions, organizational resources, and characteristics of the population in the new setting or community. Planners may not need to reject an EBI immediately if the population for the original EBI is not the same as that in the new site. Different populations or subpopulations might have sufficient characteristics in common that carrying the EBI forward to the next step would still be worthwhile. Furthermore, it is strongly recommended to develop organizational capacity rather than cutting an EBI component.

**Step 3 (assess fit and plan adaptations).**

With the materials for each candidate EBI in hand, the tasks for the third step are to (3A) judge how well the candidate EBI fits the desired behavioral and environmental conditions from the community's logic model of change; (3B) judge whether the determinants of behavior and environmental conditions and the change methods used to influence them in the original EBI are adequate in the new setting; (3C) judge how well original delivery, design features, and cultural elements fit the new setting and population; (3D) judge the fit of implementation strategies to the new setting; and (3E) consider which EBI elements are essential and decide how to retain them. If the original logic model of change and theoretical grounding are not published, planners have to work backwards from available intervention materials to try to figure out what change methods were used, which determinants were addressed, and which behaviors and environmental conditions were promoted. In addition, planners can contact the original EBI developers to obtain more information about the logic of the original EBI. Based on these assessments, planners can make a grounded selection of one EBI, and they will have a to do list of what to adapt and ideas about how to adapt. Planners should remember that less adaptation saves resources and protects an evidence-based intervention from changes that may make it less effective.

**Step 4 (modify materials and activities).**

The tasks of the fourth step are the following: (4A) prepare design documents for the adaptations and drafting changes; (4B) pretest the adapted materials; and (4C) produce final adapted materials. Design documents provide detailed descriptions of planned changes, link the change to their location in the EBI materials, and provide an outline of messages. If proposed adaptations include the* addition* of behaviors, environmental conditions, determinants, and change methods, then planners can create a separate matrix of change objectives with the behavior or environmental condition being targeted and its determinants. This matrix arrangement is described in literature about Intervention Mapping [21, 24]. IM Adapt recommends planners make sure not to make unintended changes to the logic model of the EBI when making adaptations or revising adaptations based on pretest outcomes.

**Step 5 (plan for implementation).**

The fifth step consists of the following tasks: (5A) identify implementers, implementation behaviors, and outcomes; (5B) develop implementation and maintenance scope, sequence, and instructions; (5C) plan activities to motivate and train implementers; and (5D) plan logistics including budget, staffing, and materials. Planners compare the implementation protocol of the EBI, if available, to implementation considerations and constraints for the new site to create a revised protocol. Adaptations that resulted in modifications to the program components or delivery may require a different way to implement the modified EBI. The new implementation plan should include expected implementation outcomes—delivered to whom? when? how much?—and list the persons who will implement the program and how (required implementation steps or behaviors). Next, planners specify how much of the EBI will be implemented in what sequence over what period of time and write explicit instructions for new program implementers to bring that into action. Planners explore determinants of implementation and the change methods and practical applications that would influence them. Usually, for implementation, these change methods are woven into trainings, consultation, and technical support activities [30].

**Step 6 (plan for evaluation).**

In the sixth step, planners (6A) write evaluation questions; (6B) choose indicators and measures; (6C) choose the evaluation design; and (6D) plan data collection, analysis, and reporting. The purpose of evaluating an adapted EBI is to determine whether the intervention achieves outcomes in the new setting comparable with outcomes in the original evaluation (“effect evaluation”) and whether the new setting can successfully implement the adapted EBI (e.g., by measuring reach and fidelity). Evaluation questions for adapted EBIs can be borrowed from the original EBI evaluation. If the target behavior or environmental condition has been adapted, the indicators and measures must match the new logic model.

## 3. Results: Case Study

*Step  1: Organizational Capacity, Needs Assessment, and Logic Models*

*(1A) Organizational Capacity*. A local hospital-based charity organization (the Charities) initiated the planning for the project. The Charities proposed to find an evidence-based program to reduce the no-show rate for appointments made at mobile mammography sites with primarily African American women. The planning team included representatives from the lead agency research arm, breast cancer provider organizations, the local Breast Health Collaborative (an organization to establish linkages between organizations with breast health missions), and the local school of public health. All partners had missions that encompassed improving breast health in the Houston area. The researchers from the school of public health and the Charities also had a commitment to using and evaluating evidence-based programs.

*(1B) Needs Assessment and Logic Model of the Problem*. The planning group conducted a needs assessment to examine barriers to mammography screening and appointment keeping among African American women in Houston. We present a brief summary of the assessment outcomes here, with a detailed description of methods and results previously published [14].

Research suggests that recent reductions in breast cancer mortality are related to early detection (mammography) and enhanced cancer treatment [31]. However, African American women are less likely to schedule and attend mammography screening, with appointment no-show rates in some sites of 30–50% [15–18, 31–33]. Local women described the following barriers to appointment keeping: (1) fear of the outcome (it will be cancer); (2) competing demands (taking care of everyone but myself); (3) logistical barriers, such as insurance, cost, and transportation; (4) fear of partner abandonment if mastectomy results (loss of womanhood); (5) lack of education (nobody talks about mammography/breast cancer); (6) fear the mammogram would hurt; and (7) no need for a mammogram because their faith would protect them from cancer. Next, the planning group organized the data from the needs assessment in a logic model of the problem. The planning group focused on failure to keep mammography appointments. They then included all of the information from the community data collection as determinants of the lack of mammograms.

**Figure 2:**
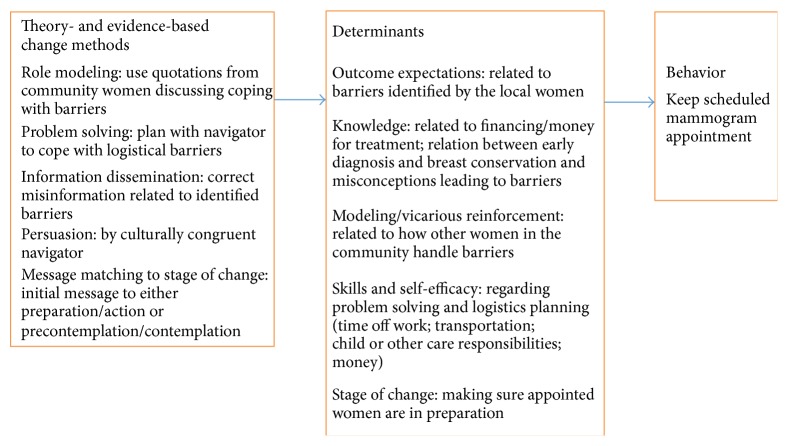
Case study logic model of change.

*(1C-D) Logic Model of Change and Intervention Goal*. Next, the planning group converted the logic model of the problem to a logic model of change to create the foundation for comparing EBIs to the intervention needs in the community (see Figure 2). The model focused on the behaviors of African American women because the environmental factors (i.e., access to treatment options and access to primary care) were not changeable in the scope of this project. The group worked from the list of local barriers to create categories of counter arguments to barriers by theoretical constructs. The barriers and counter arguments could be summarized with the following Social Cognitive Theory [34] constructs: knowledge, outcome expectations, modeling/vicarious reinforcement, skills, and self-efficacy. For example,* negative outcome expectations* such as “a diagnosis of breast cancer leads to death” could be countered by “early detection can lead to treatment and cure” and* low self-efficacy* such as “logistical problems of caring for others make mammography impossible for me to do” could be countered by “I can use the problem solving skills I use for other problems.” These theoretical category labels did not replace the natural language used by the women to describe barriers. The group completed the logic model of change by adding theory- and evidence-based change methods that are suited to influencing outcome expectations, self-efficacy, and the other determinants with methods such as persuasion, role model stories, culturally congruent role models, and guided practice for problem solving. Based on Step 1, the group set the goal to decrease missed appointments of low-income African American women by 20% in the first year of program implementation.

*Step 2: Finding an Evidence-Based Intervention*

*(2A) Searching for an EBI*. To increase the number of possible interventions found, the planning group searched for EBIs focused on improving adherence to mammograms in African American women rather than more narrowly on appointment keeping. Prior to searching, they reviewed the Community Guide [28] to understand the types of strategies recommended to improve mammography screening. The team then searched for a full intervention (one with both description and available materials) using RTIPs [14, 26]. The group found four potential candidate programs [14]. The team then performed a second search of databases of peer reviewed studies to find reports of the original evaluations conducted on the EBIs they had located.

*(2B) Assessing Basic Fit*. Using the peer reviewed articles that described the programs and their evaluations, the planning group assessed basic fit: Was the health promoting behavior the same as for the new community? Could the organizational capacity support the program? Was the program acceptable for the risk group in the new community? All of the programs fit with the goal of encouraging mammography, but no programs focused explicitly on the behavior of appointment keeping. Therefore, the group recognized that the behavioral focus had to be specified no matter which of the four candidate programs they would work with [35–38]. All of the programs targeted either multiple ethnicities or African American women and any of them might be a basic fit to the priority population. No matter the final program chosen, the planning team would need to adapt it by integrating the information from African American women about local barriers, the way they feel about and talk about mammography, and their screening intentions. The planners selected a telephone counseling program after considering program fit with the implementation capacity of the clinical partner [35]. Other programs including community, church, and home visiting programs were outside of the Charities' mission, scope, and resources.

*Step 3: Assess [Detailed] Fit and Plan Adaptations*. To judge detailed fit and plan adaptations, the planning group obtained the program manual from the original developers (available on RTIPs). The manual contained instructions and scripts for the telephone counselors to address barriers. The team reviewed each type of fit and noted planned changes on an adaptation “to do list” (see Table 1).

*(3A) Behavioral Fit*. Referencing their logic model of change, the group described adaptations required to change the behavioral focus from getting a mammogram in general to appointment keeping for women who already had appointments scheduled.

*(3B) Determinants and Change Methods*. While judging the EBI's change methods and determinants, the planning group thought that several additions should be made to the scripts. The group members could discern how the original program guided the telephone counselor to ascertain a woman's stage of change, but they were unclear about how the counselors matched change methods to stages. Therefore, the planning group decided to assess only two stage categories (precontemplation/contemplation and preparation/action) as measured by women's certainty that they would keep their appointments and then match dialogue to the stage. For example, if a woman seemed unsure (precontemplation/contemplation), the telephone counselor would explore intensively for barriers. Additionally, the planners were unclear from the manual about which determinants were targeted and recommended that Social Cognitive Theory constructs of self-efficacy, skills, and outcome expectations be added with matching change methods of persuasion, cultural congruence, role modeling, and problem solving. Finally, the group found in the manual a comprehensive list of barriers, but barriers were addressed as if most beliefs could be remedied by provision of information. The group noted that most belief change requires change methods beyond information (i.e., role models, persuasion, and guided practice).

*(3C) Delivery Fit, Design Features, and Cultural Relevance*. In this task, the planning group considered the acceptability of the EBI to the new population: whether the original delivery will reach the new population, how the design of program materials will resonate with the new participants, and how culturally congruent the entire program will feel to users. The planning group understood from the clinical partner that telephone reminders were an effective way to reach the priority population, so telephone delivery was acceptable. The group also judged that the scripts for barriers were up-to-date, accurate, and understandable but were not targeted to the exact concerns of local African American women or expressed in the ways that local women talked about their concerns. Furthermore, the original program did not outline an underlying communication approach; therefore the group agreed on an active listening framework for the scripts to maximize the connection of the navigator with the women through listening to their concerns and validating them [39].

*(3D) Implementation Fit*. Implementation fit is closely related to delivery, and the team made several adaptations to delivery as mentioned above. For instance, they decided to develop a conversational script to inquire about barriers in ways that fit with each stage category, which dealt with transitions in the conversation, and to enable the counselor to develop rapport with the women. However, implementation also has to do with how the implementing agency will manage the logistics of getting the EBI in place and maintaining it. The planning team worked with the clinical partner to plan staff placement and training for the telephone counseling. They also made sure that contact information was available for women with scheduled appointments and that data from each call and from appointment records could be recorded and accessed for the evaluation.

*(3E) Considering Essential Elements*. The planning group considered essential elements of the counseling program. The program was developed over a decade ago, and the developers were not available to answer questions. Therefore, the group independently considered the program to decide the program features that might have been essential to its effectiveness. They listed the following characteristics: (1) barrier-focused counseling (change method), (2) telephone call delivered by a person (rather than a computer) (delivery), and (3) assessment of stage of change (prerequisite for matching change methods). Looking back at their adaptation “to do list,” they made sure that their suggestions for change did not eliminate these essential program elements and sought only to enhance their intensity.

*Step 4: Make Adaptations*

*(4A) Preparing Design Documents and Adaptation Drafts*. The planning team noted the planned changes, described the program materials and activities in which the change should be made, and then wrote or edited messages that supported the change. For example, the team members proposed a foundation conversational structure based on active listening [39]. They then wrote the script to support the change. Another important adaptation was the addition of specific barriers as described by local African American women in the assessment and acknowledgement that local women had both described each barrier and also had described strategies for overcoming it (role model change method) (based on the assessment and feedback from the community advisors).

*(4B) Pretesting Adapted Materials*. Once the team adapted the program manual, it pretested the scripts with local African American women. Fourteen pretesters worked in pairs to role play the scripts with one woman as counselor and the other, as patient (caller). The pretesters noted needed changes in the scripts to make them as relevant as possible to local women. For example, they recommended taking care not to talk about a cancer diagnosis (and engender fear) in women simply being prepared to undergo screening. The pretesters then thoroughly debriefed the role plays with the entire project team. Pretesters also strongly recommended that the calls be made by a culturally congruent counselor.

*(4C) Producing Final Adaptations*. The team produced the revised manual of barriers and foundational conversation scripts organized in hard copy form. The hard copy format was used for the initial implementation and evaluation of the adapted EBI [40]. Following the initial evaluation study, the manual was converted to computer-assisted scripts for use by a live counselor [19].

*Step 5: Plan for Implementation*

*(5A) Identifying Implementers, Behaviors, and Outcomes*. The mammography team found the implementation protocol for the original program (prepared for research staff rather than patient counselors) to be focused on assessment of stage of change and barriers with little guidance to the implementer about how to transition from assessment to barriers and how to transition between barriers. The original manual was helpful, but it was not sufficient for this new site. Therefore the team identified implementers as patient navigators or community health workers familiar with making reminder calls and with identified implementation tasks or behaviors including (1) making standard reminder calls; (2) making protocol-driven, barrier-focused counseling calls for African American women already appointed for a mammogram; and (3) documenting the content of each call. Other implementers were the clinical partner managers who would provide space and access to appointment records for the navigator. The partner would also provide data on patient appointment attendance for the evaluation study. The desired implementation outcome was the completion of at least 100 EBI calls and 100 standard reminder calls in twelve months.

*(5B) Developing Scope, Sequence, and Instructions*. The scope of the adapted EBI was one call per woman. The sequence was seen as the sequence of the call to include assessment of stage, query regarding barriers, and solutions to barriers based on barrier scripts. The technique for moving the conversations forward was based on active listening [39].

*(5C) Planning Activities to Motivate and Train Implementers*. The mammography team developed training to encourage self-efficacy, outcome expectations, and skills of the implementers. The skills included opening, moving, and closing conversations, establishing rapport, conducting active listening, and addressing barriers. In the training sessions, we explained the theory behind the program but spent the majority of the time in the sessions conducting role play practice with feedback.

*Step 6: Plan for Evaluation with a Focus on Adaptations*. The evaluation plan sought to accomplish two aims: (1) determine the effectiveness of the adapted EBI in improving appointment keeping for mammography in African American women and (2) describe processes of implementation of an EBI in a practice setting. For evaluation results, see Highfield et al. in this issue [20].

*(6A) Writing Evaluation Questions*. We wrote the following evaluation questions for the effectiveness evaluation: (1) What was the effectiveness in decreasing appointment “no-show” rates in the new setting? (2) How did the effectiveness of the adapted EBI compare to the effectiveness of the original EBI? The questions for implementation evaluation (process) included the following: (1) Was the adapted EBI delivered to the intended population (i.e., low-income African American women with mobile mammography appointments)? (2) Did the implementers follow the protocol (i.e., implemented with fidelity?)? (3) What barriers were discussed in the phone calls? (4) Did the women who received the adapted EBI find it helpful and acceptable? (5) What problems occurred during implementation of the adapted EBI?

*(6B) Choosing Indicators and Measures*. To measure effectiveness, we obtained kept and missed appointments from the electronic database of the clinical partner. We also collected site of mammography, time between phone call and appointment, age, date, and time of appointment, counselor information, and contact information including phone number. We compared intervention phone calls to the protocol (whether the navigator asked the staging question, used the barrier scripts, conducted logistical planning, and used active listening). In addition, we interviewed randomly selected intervention patients regarding their perceptions of the EBI calls and systems barriers encountered.

*(6C) Choosing the Evaluation Design*. We used the type-1 hybrid design to test the intervention's effectiveness and to gather information on the implementation [40, 41]. We used a quasi-experimental, sequential recruitment design in which we assigned contacted women to usual care or adapted intervention in groups of 50 patients.

*(6D) Planning Data Collection, Analysis, and Reporting*. We enrolled African American females who were aged between 35 and 64, uninsured, and had income of ≤200% of the federal poverty level (FPL) and with an upcoming appointment for a mobile screening mammogram. We tracked all data for the pilot either in an access database or in paper data collection forms. We calculated descriptive statistics and then conducted logistic regression analysis to report attendance in the intervention group as compared to the comparison group while controlling for potential confounders. Following the basic analysis, we further evaluated the effectiveness of the EBI using intent to treat analysis [41–46].

### 3.1. Project Outcomes and Current Status

The evaluation for this project is completed and the results have been used to acquire funding for a larger implementation of the adapted EBI [19, 20]. For the evaluation results, see Highfield et al. [20]. The effectiveness results were in the range of the results from the original intervention evaluation and indicated improved EBI effectiveness as a result of the systematic adaptation process [20, 35]. The implementation evaluation allowed us to discover problems in the initial implementation and correct them with a change in evaluation design and eventually in personnel.

## 4. Discussion

IM Adapt provides a systematic “how to” process to guide intervention adaptation, implementation, and evaluation. While we illustrate the approach using a mammography appointment attendance EBI, the processes used and described in this case study are widely applicable for the adaptation of any EBI. Intervention Mapping has been used worldwide to help planners develop and implement EBIs for a variety of public health problems including cancer prevention (cervical and breast), nutrition, parent education to reduce violence, and asthma, to name a few [47–52]. Other researchers and planners have used models for guiding EBI adaptation, such as Planned Adaptation [10]. For example, in their study of Planned Adaptation, Lee et al. noted that future studies should explicitly consider the roles of practitioners in participating in EBI translation. It has also been noted that, for cancer disparities in particular, EBIs should be adopted and tailored at the community level by partnerships that include both researchers and practitioners, adding an additional level of complexity to the translation of these EBIs [53]. IM Adapt specifically incorporates practitioners throughout the adaptation process and gives them a structured role through the planning team.

Additionally, Lee et al. [10] noted the need for models of adaptation to guide users through the process of developing appropriate evaluation and measurement plans. IM Adapt addresses this gap through having users consider evaluation questions that need to be addressed. Additionally, the IM Adapt process guides planners to incorporate both effectiveness and process evaluation. Curran et al. [40] have suggested the need for blended EBI effectiveness and implementation trials that consider both effectiveness outcomes and implementation process [40]. Blended effectiveness/implementation trials are a key area of study which are currently underreported [2, 40, 54]. As noted by Wandersman et al., 2008 [55], publication of these kinds of studies is necessary to guide future efforts to disseminate EBIs into practice. Practitioners lack sufficient insight into this process and as a result are forced to make decisions based on limited information [24].

### 4.1. Limitations of the IM Adapt Process and of the Case Study Project

IM Adapt helps planners compare their local health problem and needs to available EBIs to judge fit of the intervention in terms of the health problem and its causes and then to continue the comparison to the logic model of change. This is a fairly complex process that is made more difficult by inadequate reports of EBIs in the scientific literature. Often original investigators neither publish their logic model of the problem (evidence and assumptions about the problem at the developing site of the EBI) nor explicitly address the logic model (theory) of change. This situation leaves the adopter of an EBI sometimes peering into a black box and making guesses about what change methods are contained in a program, what determinants they were meant to influence, and whether original investigators consider them to be essential program elements that should not be adapted. Improving intervention reporting would significantly improve the ability of those who want to use EBIs to choose one, decide whether adaptations are advisable, and use a systematic process to carry them out if they are needed. We applied the IM Adapt process to a case study of an adapted mammography appointment adherence EBI. Future studies are necessarily applying IM Adapt to a variety of EBIs and public health issues to allow for continued evaluation and refinement of the approach.

## 5. Conclusion

In this report, we have presented a case study of a community project for which we used the IM Adapt framework to find, adapt, implement, and evaluate an EBI to help underserved African American women in Houston, Texas, keep appointments for mammography screening. IM Adapt should be useful for planners who are considering EBIs to avoid developing an intervention from the beginning. Not wanting to develop an intervention de novo can be from awareness of insufficient resources for developing and evaluating a theory- and evidence-based intervention or because a funder has required the use of an existing EBI. If a planning group is able to find an EBI that addresses its priority health problem, it will face a core question, does this program fit with our community and with the characteristics of the health problem in the new setting and can it be adapted so that it better fits and still works? IM Adapt is a guide for answering this question and performing a systematic adaptation.

## Figures and Tables

**Table 1:** Adaptation “to do list” for telephone counseling program.

Breast Cancer Screening Among Nonadherent Women	

Fit category	Adaptation ideas

*Behaviors from logic model of change* Adherence to mammography	Change behavior to “appointment keeping” rather than general mammogram

*Environmental conditions from logic model of change*	No change

*Change methods (with determinants) for at risk group*: Information dissemination (barriers) Staging (but does not seem closely related to change methods beyond information)	Role modeling: quotations from women in the community regarding barriers Problem solving: regarding logistical barriers Information dissemination: correcting misinformation Persuasion: by culturally congruent navigator Message matching to stage of change: initial message to either preparation/action or precontemplation/contemplation

*Change methods (with determinants) for environmental agents*	Not applicable

*Delivery for components*,* at risk group*: Telephone counseling call. Note: does not have conversational structure comfortable for navigators to implement	Change staging question and scripts to be less research oriented and more “real-world” navigator approach Develop an active listening framework for barrier scripts Retain staging but with only two classifications (precontemplation/contemplation; preparation/action) Editing for local idiom (ways of speaking of breast cancer and barriers)

*List delivery for components, environmental agents*	Not applicable

*List design features and cultural relevance* Barriers are general and information-based	Add barriers described by local women: perceived likelihood of no cancer; expectation that God will protect against cancer; no money for treatment; becoming less than a woman with the loss of a breast; fear of losing partner; cancer being a death sentence; time only for caring for others Logistics: no time off work; no transportation; responsibilities caring for a child or others; money/lack of awareness of programs that can pay for breast cancer treatment

*Describe implementation plan* Research-based; elaborate staging; unclear how script changes based on staging; unclear transition between one barrier and the next	Add script with conversational transitions Add local barriers Simplify stages Match script to stage
